# Perinatal Dioxin Exposure and Attention Deficit Hyperactivity Disorder (ADHD) Symptoms in Children Living in a Dioxin Contamination Hotspot in Vietnam

**DOI:** 10.3390/toxics10050212

**Published:** 2022-04-24

**Authors:** Tai Pham-The, Muneko Nishijo, Thao Ngoc Pham, Hoa Thi Vu, Nghi Ngoc Tran, Anh Hai Tran, Luong Van Hoang, Quyet Do, Yoshikazu Nishino, Hisao Nishijo

**Affiliations:** 1 Institute of Biomedicine and Pharmacy, Vietnam Military Medical University, Hanoi 12108, Vietnam; phamngocthaovmmu@gmail.com (T.N.P.); anhhtr@yahoo.com (A.H.T.); hoangvanluong2003@yahoo.com (L.V.H.); dobaquyet@yahoo.com (Q.D.); 2 Department of Public Health, Kanazawa Medical University, Uchinada, Ishikawa 920-0293, Japan; ni-koei@kanazawa-med.ac.jp (M.N.); vuhoa5593hvqy@gmail.com (H.T.V.); ynishi-no@kanazawa-med.ac.jp (Y.N.); 3 Ministry of Health, Vietnamese Government, Hanoi 10060, Vietnam; nghi_tranngoc@yahoo.com; 4 Faculty of Medicine, University of Toyama, Toyama 930-0194, Japan; nishijo@med.u-toyama.ac.jp

**Keywords:** dioxins, birth cohort, attention deficit hyperactivity disorder (ADHD), autistic traits, sex difference, age difference

## Abstract

We examined children in Da Nang, a dioxin contamination hotspot in Vietnam, twice at 5 and 8 years of age, and investigated sex- and age-dependent differences in the effects of dioxin exposure on attention deficit hyperactivity disorder (ADHD) symptoms. We also studied autistic traits in children with ADHD symptoms. A total of 163 children participated in follow-up surveys at 5 and 8 years of age and were included in the present analysis. ADHD symptoms were assessed using an ADHD rating scale with inattention and hyperactivity-and-impulsivity (hyperactivity) subscales. Autistic behaviors were evaluated using the Autism Spectrum Rating Scale (ASRS). Perinatal dioxin exposure was indicated by dioxin levels in maternal breast milk. In boys, hyperactivity scores were significantly higher in the high 2,3,7,8-tetrachlorodibenzo-p-dioxin (TCDD) group only at 5 years of age. In girls, hyperactivity scores at 8 years of age were significantly higher in the high TCDD group, which was significantly associated with those at 5 years of age. In girls, ASRS unusual behavior scores were significantly higher with higher TCDD exposure and hyperactivity scores at 8 years of age. These results suggest that high perinatal TCDD exposure may increase ADHD likelihood and autistic traits, particularly in girls of 7–8 years of age.

## 1. Introduction

Endocrine disrupter chemicals (EDCs), such as polychlorinated biphenyls (PCBs), are suggested to have important roles in increasing the prevalence of neurodevelopmental disorders and behavior problems in children exposed to EDCs during the perinatal period [1]. Attention deficit hyperactivity disorder (ADHD) is one of the behavioral problems of which an increase has been of public concern in recent years [1]. Thus, associations between ADHD and EDCs, including PCBs [2,3,4,5], pesticides [6,7], and lead [8,9,10] exposure, have been investigated in contaminated areas in the United States and all over the world. However, there are not many previous studies investigating associations between ADHD symptoms and dioxins, which are among the most common EDCs [1], except for two birth cohort studies in dioxin-contaminated areas in Germany [11] and Italy [12].

From 2008 to 2009, we followed a birth cohort living in a dioxin-contaminated area in Da Nang, Vietnam, near a former U.S. military airbase, and investigated the effects of perinatal dioxin exposure on growth and neurodevelopment of children at various ages. Previously, we reported that lower planning ability associated with increasing toxic equivalency values of polychlorinated dibenzodioxins and polychlorinated dibenzofurans (TEQ-PCDD/Fs) in boys at 5 years of age [13]. In 8-year-old children, increased dioxin exposure was significantly associated with poor learning ability indicated by higher Colorado Learning Difficulties Questionnaire (CLDQ) scores, particularly in boys [14]. These results suggest that boys are susceptible to the effects of dioxin and their neurodevelopment was poorer than that of girls.

Regarding emotional and behavioral disorders, we reported increased children with autism spectrum disorder (ASD) behaviors associated with perinatal 2,3,7,8-tetrachlorodibenzo-p-dioxin (TCDD) exposure at 3 years of age for both sexes (boys are more likely than girls) [15]. In the survey of 8-year-olds, aggressive behavior was also examined by interviewing parents or caretakers of children using the Children’s Scale of Hostility and Aggression: Reactive/Proactive (C-SHARP) with five subscales (verbal aggression, bullying, covert aggression, hostility, and physical aggression). The prevalence of high covert aggression scores in children of both sexes was significantly higher with higher TCDD exposure with an adjusted odds ratio of 4.5 [16]. However, in stratified analysis according to sex, the significant association between covert scores and TCDD exposure was clearer in girls than in boys [17]. When aggressive behavior is often observed in people with ADHD, it was suggestive and necessary to clarify the effects of dioxin on ADHD in this children cohort.

Furthermore, children with ASD often show inattention and impulsivity-hyperactivity and are diagnosed with ADHD as a comorbidity of ASD [18] because ASD and ADHD are reported to share etiological factors, such as inflammation in the brain [19,20] and disrupted gut microbiota that increases intestinal inflammation [21,22]. Hence, the aim of this present study was to investigate the associations between perinatal dioxin exposure and ADHD symptoms at different ages and sexes. In addition, the relationships between autistic behaviors and ADHD symptoms were analyzed to investigate increased autistic traits in children with increased ADHD symptoms.

## 2. Materials and Methods

### 2.1. Study Area and Subjects

Thanh Khe and Son Tra districts in Da Nang city were chosen as the study areas. These districts are located within 10 km of the former Da Nang airbase and are dioxin contamination hotspots originating from herbicide spraying in Vietnam. The subjects were children from the Da Nang birth cohort, including 238 mother-and-infant pairs (158 pairs in Thanh Khe and 80 pairs in Son Tra), recruited in 2008–2009 and followed up from 1 month to 8 years old [13,14,15,23,24,25].

A total of 168 mother–infant pairs (71.4% of birth cohort; 97 boys and 71 girls) participated in the follow-up surveys at 5 and 8 years of age. Because of missing behavioral examination data, 163 children (94 boys and 69 girls) were enrolled in the present analysis.

### 2.2. Perinatal Dioxin Exposure Levels Indicated by Dioxins in Breast Milk

The children’s perinatal dioxin exposure was estimated using the dioxin levels in their mothers’ breast milk collected a month after the birth of these children. Seventeen 2,3,7,8-substituted congeners of polychloro-dibenzodioxins (PCDDs) and polychloro-dibenzofurans (PCDFs) were measured using a high-resolution mass spectrometer (MStation-JMS700, JEOL, Tokyo, Japan), and the total toxic equivalents (TEQ) of PCDDs and PCDFs (TEQ-PCDD/Fs) were calculated by summing all of the values obtained by multiplying each congener concentration with reference to the WHO 2005 toxic equivalent factor [26]. The established method of analysis has been described previously in detail [27]. Because TCDD is the most toxic dioxin congener and specific for dioxin contamination originated from Agent Orange and TEQ-PCDD/Fs reflected the total toxic equivalent of all 17 PCDD/Fs, we selected these 2 indices as dioxin exposure markers in the present study.

### 2.3. Behavioral Assessment

The attention deficit hyperactivity disorder rating scale (ADHD-RS): parent ratings (2–5 years) was used to evaluate the children’s behavioral disorders by parents or caretakers when children reached 5 and 8 years of age. The ADHD-RS includes two subscales, an inattention score (inattention) and impulsivity and hyperactivity (hyperactivity) score and a total scale score (ADHD). The values of each scale were standardized by age and sex with a reference to percentiles in the score sheets with the range of 1 to 99 percentiles [28]. Since this scale has no Vietnamese version, we translated this scale from the original version in English into Vietnamese and conducted a trial examination with 12 Vietnamese children to ensure the feasibility and appropriateness of this scale for the Vietnamese population.

Autistic behavior was evaluated using autism spectrum rating scales (ASRS) Autism Spectrum Rating Scales™ (ASRS**^®^**); Multi Health Systems Inc., (North Tonawanda, NY, USA) with three subscales, social communication (SC), unusual behavior (UB), and Diagnostic and Statistical Manual for Mental Disorders (DSM), and total scale (TOT) after percentile rank conversion from raw values to T-scores with the range of 25 to 85 using the technical manual for ASRS™ [29]. This scale has been translated from the English version into Vietnamese and applied to 179 3-year-old children from the Da Nang cohort [15].

Both the ADHD-RS and ASRS were standardized for children in the United States and not for those in Vietnam. Therefore, we cannot diagnose or estimate a risk of ADHD and ASRS for individual children based on the test results. However, a single examiner interviewed all participants for each scale to make scores reliable enough for indicating ADHD or ASD traits within group.

### 2.4. Statistical Analysis

SPSS (version 21.0) for Windows (IBM Corp., Armonk, NY, USA) was used to perform statistical analyses. TCDD concentrations and TEQs in breast milk were base-10 logarithmically transformed to normalize data distribution. Basing on the previous publications regarding the possible effective benchmarks of dioxin levels in this cohort, the cutoff values for high and low TCDD and TEQ-PCDD/Fs groups were set as 3.0 pg/g lipid and 17.6 pg-TEQ/g lipid, respectively. These cutoff values were derived from the 88th percentile concentration of TCDD and the 75th percentile level of TEQ-PCDD/Fs in breast milk [17,30].

General linear models were used to compare the mean ADHD-RS and ASRS scores between high and low TCDD or TEQ-PCDD/Fs groups after adjusting for covariates, including age, parity, maternal education, family income, gestational weeks, birth weight, and age in months at examination of children. Spearman’s rho was used to analyze correlations between ADHD-RS scores at 5 and 8 years of age and between ADHD-RS scores and ASRS scores.

## 3. Results

### 3.1. The Characteristics of Mother-Child Pairs and Dioxin Exposure Levels

The characteristics of mothers and gestational weeks and weight at birth and age in months and body size at the time of the survey at 5 and 8 years old are shown in Table 1. There was no significant difference in the characteristics of mothers and children at birth between sexes. However, the mean BMI was significantly higher in boys at 5 and 8 years old compared with girls (*p* < 0.05 for *t*-test).

The geometric means with geometrical standard deviations of 17 PCDD/Fs congeners and TEQ-PCDD/Fs in maternal breast milk are shown according to child sex in Table 1. There was no significant difference of means for any congeners nor TEQ-PCDD/Fs between sexes. The mean TCDD of these samples was around three times higher than that in the unsprayed area (0.6 pg/g lipid) [27]. In addition, the mean TEQ-PCDD/Fs was four times higher than that in the unsprayed area (3.7 pg-TEQ/g lipid).

### 3.2. Dioxin Exposure and ADHD-RS Scores at 5 and 8 Years of Age

For each sex, ADHD-RS scores for inattention, hyperactivity, and ADHD were examined at 5 years of age and compared between high and low TCDD and TEQ-PCDD/Fs exposure groups (Table 2). In boys, hyperactivity and ADHD scores were significantly higher in the high TCDD group (≥3 pg/g lipid) than in the low TCDD group (<3). In girls, the hyperactivity scores and ADHD scores in the high TCDD group were more than 10 points higher than those in the low TCDD group; however, the differences between groups were not significant. For TEQ-PCDD/Fs, there was no significant difference in any ADHD-RS scores between the high and low exposure groups at the cut-off value of 17.6 (pg-TEQ/g lipid) in either sex (Table 2).

Three ADHD-RS scores, inattention, hyperactivity, and ADHD scores, examined in the 8-year-old survey were also compared between high and low TCDD and TEQ-PCDD/Fs groups, and the results for each sex are shown in Table 3. In boys, there was no significant difference in any ADHD-RS scale scores between high and low TCDD and TEQ-PCDD/Fs groups. Whereas, in girls, hyperactivity scores in high TCDD and TEQ-PCDD/Fs groups were significantly higher than those in low exposure groups, respectively.

### 3.3. Relationship between ADHD-RS Scores at 5 and 8 Years of Age

Higher ADHD-RS scores associated with increasing dioxin exposure were found in boys at 5 years of age and girls at 8 years of age, and therefore, Spearman’s correlations between the ADHD-RS scores at 5 years and 8 years of age were analyzed in high and low TCDD groups and all children for each sex (Table 4). In the low exposure group and all children, almost all ADHD-RS scores at 8 years of age significantly correlated with those at 5 years of age for both sexes, except for the inattention scores in 8-year-old girls. However, in the high TCDD group, there was no correlation of any scale scores at 5 and 8 years of age in boys, while hyperactivity scores at 8 years of age significantly correlated with hyperactivity and ADHD scores at 5 years of age in girls.

### 3.4. Dioxin Exposure and ASRS Scale Scores at 5 Years of Age

In the survey at 5 years old, we also examined autistic traits in children using the ASRS scale and compared the mean SC, UB, DSM, and TOT scores between high and low dioxin exposure groups for each sex (Table 5). At this time, the ASRS scores for three boys and three girls were missing because there was no sufficient reply from these children’s caretakers. The adjusted UB scores were significantly higher in the high TCDD group than in the low TCDD group only in girls. However, no significant difference in any ASRS scores between high and low TEQ-PCDD/Fs groups was observed in either girls or boys.

### 3.5. Relationship between ASRS and ASHD-RS Scores at 5 and 8 Years of Age

To clarify the relationship between the ADHD-RS and ASRS scores, the Spearman’s correlation between ADHD scale and ASRS scale scores at 5 and 8 years of age was analyzed (Table 6). Significant correlations were found between all ADHD-RS scores at 5 years of age and UB scores in both sexes. In addition, in boys, inattention and ADHD scores also correlated with TOT scores. At 8 years of age, inattention and ADHD scores significantly correlated with TOT scores in boys. However, in 8-year-old girls, only the hyperactivity scores significantly correlated with UB scores.

## 4. Discussion

### 4.1. Perinatal Dioxin Exposure and ADHD Symptoms in Children Living in a Dioxin Contamination Hotspot in Vietnam

In the present study, increased ADHD symptoms were observed in children perinatally exposed to dioxins at higher levels than 3 (pg/g lipid) for TCDD and 17.6 (pg-TEQ/g lipid) for TEQ-PCDD/Fs compared with less exposed children. These exposure levels were similar to those which showed neurodevelopmental deficits in our previous studies for children from the Da Nang cohort [13,14,15]. However, dioxin effects on ADHD symptoms were clearer in girls than those in boys, in contrast with the studies of the same subjects (boys > girls) that targeted on other aspects of neurodevelopment such as motor coordination and higher cognitive development [13] and learning abilities in school [14].

In boys, hyperactivity and ADHD scores at 5 years of age were significantly higher in the high TCDD group than in the low TCDD group. However, we could not confirm the presence of increased children with ADHD traits based on these findings because 5 years old is not the eligible age for ADHD diagnosis (7–8 years old) with reference to the ADHD criteria in the DSM fourth edition [31].

In contrast, hyperactivity scores in girls at 8 years of age were significantly higher in the high TCDD and TEQ-PCDD/Fs groups than in the low exposure groups. In addition, in girls, hyperactivity scores at 8 years of age were significantly associated with those at 5 years of age, suggesting that ADHD symptoms, particularly impulsivity-hyperactivity, are increased at preschool age and reached levels sufficiently high to diagnose hyperactive-impulsive-type ADHD (ADHD-PH) in early school age (DSM fourth edition criteria). However, these results are inconsistent with two epidemiological facts in unexposed children with ADHD: (1) ADHD is more frequently found in boys and (2) a predominantly inattention type (ADHD-PI) is more common than ADHD-PH in girls [32]. These differences in ADHD characteristics between dioxin-exposed and non-exposed children might be related to the endocrine disrupter effects of dioxins, particularly TCDD. Moreover, the small number of subjects in high exposure group (n = 9 for boys) may decrease the statistical power in comparisons. Further large-scale studies from the perspective of sex differences are necessary in adolescents to clarify this issue.

In the present study, we analyzed child behavior based on a parent rating scale and did not examine their performance using neuropsychological function tests, such as continuous performance tasks (CPTs), which are commonly used to examine children with ADHD. Children with ADHD often have impaired neuropsychological functions, including vigilance, working memory, and response inhibition, which are related to their characteristic ADHD symptoms [32]. Therefore, in the future, CPTs should be performed in children to examine the neurobehavioral impairments associated with dioxin exposure and compare with those in non-exposed children with ADHD.

### 4.2. PCB and Dioxin Exposure and ADHD Symptoms in Children in Countries Other Than Vietnam

The WHO has carried out a series of exposure studies on the levels of PCDDs, PCDFs and PCBs in breast milk. The first WHO-coordinated exposure study took place in 1987–1988, the second round in 1992–1993, and the third round in 2000 to 2003 [33]. Temporal trends in the levels of PCDDs and PCDFs in human milk for countries around the world was observed continuously. For example, the decline between the levels found in the second round in 1993 and those found in the third round in 2003 is about 40%. In the third round, the lowest levels of PCDDs/PCDFs were found in countries in the Southern hemisphere (Fiji, Brazil, Philippines, Australia), from 3.34 to 5.57 pg-TEQ/g lipid, whereas the levels of PCDDs/PCDFs in European countries (Bulgaria, Croatia, Hungary, Ireland, Ukraine, Italy, Spain, Germany, Luxembourg) were comparatively high, from 6.14 to 14.97 pg-TEQ/g lipid. The countries in the group with the highest PCDD/Fs level included Belgium (16.92 pg-TEQ/g lipid), the Netherlands (18.27 pg-TEQ/g lipid), and Egypt (22.33 pg-TEQ/g lipid) [33,34]. In our present study, the level of TEQ- PCDDs/Fs was approximately 13 pg-TEQ/g lipid (Table 1), which is in the range of some European countries. The cutoff value for division into the low and high exposure groups was 17.6 pg-TEQ/g lipid, which was equal to the levels of countries in the highest group.

Associations between cord blood PCB and impulsivity examined using CPTs were reported at 4, 8, and 9 years of age in children in a birth cohort study in Oswego, USA [2,3]. A significantly increased risk of ADHD behavior indicated by Conner’s teacher rating index in children with the highest quantile of PCB in cord blood was reported in children at 8 years of age whose mothers were residing in areas near a PCB-contaminated harbor in New Bedford, USA [4]. Sagiv et al. (2012) also examined these children using CPTs and showed significant associations between high rates of errors of omission and PCB exposure only in boys, suggesting sex-specific effects of PCBs on vigilance, an attention function [5].

In the Korean general population aged 12–15 years in the National Health and Nutrition Survey, a significantly increased prevalence of attention deficit disorder associated with increasing levels of serum dioxins, including HpCDD, OCDD, and HpCDF, was reported [35]; however, no rating scales or performance tests were used to estimate prevalence. In Germany, Neugebauer et al. (2015) examined attention in children aged 8–9 years from the Duisburg birth cohort exposed to dioxins and PCBs using a computer-based test battery KITAP and reported that significantly increased omission errors in divided attention were associated with increasing maternal blood levels of TEQ-PCBs and TEQ-PCDD/Fs [11]. However, they also reported conflicting results where parent rating ADHD scores were inversely associated with TEQ-PCBs levels in the same study subjects, suggesting no clear association between dioxin and PCB exposure and ADHD symptoms.

In children aged 7–17 years included in the Seveso second generation study in Italy from 2014 to 2016 whose mothers were exposed to extremely high levels of TCDD due to an industrial explosion in 1976, neurophysiological functions associated with attention and hyperactivity in children were examined using CPTs [12]. However, associations between prenatal TCDD exposure reflected by maternal serum TCDD collected in 1976 and ADHD index was found only in the children who were breastfed within 1 month [12], indicating no clear evidence of TCDD exposure on ADHD behavior.

In summary, these results from previous studies in countries other than Vietnam suggest perinatal EDCs exposure; for example, PCBs may increase children with ADHD symptoms at 8–9 years old [2,3,4,5,6,7,8,9,10]. However, the effects of perinatal dioxin exposure, particularly TCDD, which is the most toxic congener of dioxins [26], on ADHD symptoms and behavior were unclear in the dioxin studies [11,12] because there was no display of TCDD measurement values in the maternal samples during the perinatal period. We believe our birth cohorts in Vietnam are unique populations exposed to TCDD during perinatal period, which can provide more evidence for the effects of TCDD on neurophysiological function and behavior. Therefore, in the future, we need to conduct more studies with large populations with a variety of exposure levels in Vietnam to clarify the effects of TCDD on ADHD symptoms and behavior.

### 4.3. Associations between ASD and ADHD Symptoms

We previously reported increased autistic traits as reflected by ASRS scores in children with perinatal exposure of TCDD ≥ 3.5 (pg/g lipid) in both sexes (boys > girls) when children from the present birth cohort reached 3 years of age [15]. In the present study at 5 years of age, children with perinatal TCDD exposure ≥ 3.0 (pg/g lipid) also showed increased autistic traits. However, significantly increased scores were limited to the UB subscale and found only in girls. These differences in current ASRS examination results at 5 years of age from those at 3 years old may be partly caused by the lower cut-off value of the exposure group in the present study, meaning that the high TCDD group included moderately affected children, particularly with respect to boys. Moreover, some boys whose ASRS scores were high at 3 years of age might have acquired communication skills after the survey, resulting in no more typical autistic symptoms at 5 years of age. However, ADHD symptoms at 5 years of age significantly correlated with ASD-related unusual behaviors, suggesting that boys still have mildly increased autistic traits, which may increase ADHD-like behaviors at a preschool age.

In contrast, in girls, UB scores, which were significantly higher in the high TCDD group, significantly correlated with hyperactivity scores at 5 and 8 years of age. These results suggest that high TCDD exposure may increase behavioral disorders in the form of combined ASD and ADHD, which became clear at an early school age. For these children at 9 years of age, we also reported that TCDD exposure may influence the EEG power of the mirror neuron system of the brain, particularly in girls, which contributes to social-emotional behavior and is often found in children with ASD [30]. These neurophysiological findings support that girls exposed to high levels of TCDD may have atypical brain development, leading to combined ASD and ADHD, which has more notable symptoms than those of ASD alone at an early school age.

### 4.4. Associations between ADHD Symptoms, Learning Ability, and Aggressive Behavior

In boys aged 8 years, the Colorado Learning Difficulties Questionnaire (CLDQ) reading scores were significantly higher and reading errors of some passages in the high TCDD exposure group suggested learning difficulties at school [10]. However, the C-SHARP aggression examination also showed no increase in any type of aggression and there was no change in ADHD-RS scores in boys [17], suggesting boys with high TCDD exposure may show poor performance at school but had no active problematic behaviors, such as ADHD and aggressive behaviors.

In contrast, in girls, an increased covert aggression score was significantly associated with higher TCDD levels and ADHD-RS scores [17]. However, no difference in CLDQ scores and language achievement scores was found between high and low TCDD groups in girls at 8 years of age [14], suggesting no influence of dioxins on learning ability and school performance in girls. In our previous studies on neurodevelopment in children from the present Da Nang cohort from 4 months to 5 years old, the effect of dioxin exposure on neurodevelopmental indices, including cognitive, linguistic, and motor abilities including co-ordination motor skills, was found only in boys [13,14,23,24].

Anxiety and depression are common comorbidities in children with ADHD [32]. In future, we will follow-up these children into adolescence over 12 years of age to examine their mental health status using anxiety and depression scales, as well as the ADHD-RS and C-SHARP aggression scale, to clarify dioxin effects on social-emotional behavior in these children from Vietnamese birth cohorts.

### 4.5. Strength and Limitations

Our study reported increased children with ADHD associated with dioxin, particularly TCDD, exposure during the perinatal period. To our knowledge, this is the first study to report a significant association between perinatal TCDD exposure indicated by dioxin levels in maternal breast milk and ADHD. Similarly, previous studies in the USA reported the impact of prenatal PCB exposure on the increased prevalence of ADHD in children [2,3,4]; however, their exposure levels were assessed using PCB levels in the cord blood, which shows the levels to which the fetus was directly exposed. Despite this limitation of not assessing exposure levels, we have nevertheless shown good correlations between dioxin concentrations, particularly TCDD, in breast milk and cord blood [36], indicating that TCDD in breast milk can be a good exposure marker during the fetal period.

In addition, the sample size of high TCDD group, particularly in boys, is a limitation of the present study that decreases the statistical power in comparisons of ADHD-RS and ASRS scores between high and low exposure groups. In addition, we have no control group, which should be enrolled from unexposed populations in unsprayed north Vietnam. However, they have very different maternal education levels and economic statuses from those in Da Nang located in south Vietnam, which are too strong confounding factors for adjusting associations between dioxin exposure and increased children with ADHD.

Another limitation is the ADHD-RS, which is based on the ADHD criteria in the DSM fourth edition, which is an older version than the DSM fifth edition [37] used at present time. Furthermore, performance tests, such as CPTs, were not performed in the present study, and they should be used to obtain objective results in future.

## 5. Conclusions

Perinatal TCDD exposure temporally increased hyperactivity-and-impulsivity in boys only at a pre-school age (5 years). In contrast, in girls, TCDD exposure increased hyperactivity-and-impulsivity at an early school age (8 years) and unusual ASD behavior at a younger age. These results suggest that school-aged girls perinatally exposed to high TCDD levels are more likely to show ADHD symptoms combined with autistic traits than boys of the same age.

## Figures and Tables

**Table 1:** Characteristics of the subjects and dioxin exposure in maternal breast milk.

	Boys (N = 94)		Girls (N = 69)	
Characteristics	Mean, [N]	SD, (%)	Mean, [N]	SD, (%)
*Mothers*				
Age (years)	28.2	5.9	28.8	6.6
Parity (% of primiparae)	[25]	(26.6)	[21]	(30.4)
Education (years)	8.7	3.3	8.4	3.6
Alcohol consumption during pregnancy (%)	[14]	(14.9)	[13]	(18.8)
Smoking habit of family members (%)	[82]	(87.2)	[59]	(85.5)
Family income (millions VNDs/month)	3.0	1.5	3.0	1.8
*Children*				
Gestational age (weeks)	39.6	0.8	39.6	0.8
Birth weight (g)	3259	395	3179	373
At the 5-year survey				
Age (months) at the survey	62.1	1.7	62.0	1.8
Weight (kg)	20.0	4.4	18.1	3.3
Height (cm)	109.1	4.5	107.9	4.1
BMI	16.7	2.6	15.5	2.1
At the 8-year survey				
Age (months) at the survey	92.8	1.4	93.1	1.5
Weight (kg)	28.0	7.1	24.2	4.9
Height (cm)	124.9	5.4	124.1	4.5
BMI	17.7	3.3	15.7	2.7
Dioxins in maternal breast milk *				
2,3,7,8-TCDD (pg/g-lipid)	1.4	2.0	1.5	2.6
1,2,3,7,8-PeCDD (pg/g-lipid)	4.3	1.6	4.3	1.7
1,2,3,4,7,8-HxCDD (pg/g-lipid)	2.3	1.6	2.4	1.6
1,2,3,6,7,8-HxCDD (pg/g-lipid)	8.4	1.6	8.4	1.8
1,2,3,7,8,9-HxCDD (pg/g-lipid)	2.7	1.6	2.7	1.7
1,2,3,4,6,7,8-HpCDD (pg/g-lipid)	12.1	1.5	12.5	1.6
OCDD (pg/g-lipid)	69.1	1.6	70.5	1.6
2,3,7,8-TCDF (pg/g-lipid)	0.5	1.9	0.5	2.2
1,2,3,7,8-PeCDF (pg/g-lipid)	1.2	1.7	1.3	2.0
2,3,4,7,8-PeCDF (pg/g-lipid)	7.2	1.5	7.5	1.7
1,2,3,4,7,8-HxCDF (pg/g-lipid)	17.0	1.7	18.9	1.8
1,2,3,6,7,8-HxCDF (pg/g-lipid)	10.5	1.7	11.7	1.8
1,2,3,7,8,9-HxCDF (pg/g-lipid)	0.2	2.2	0.3	2.6
2,3,4,6,7,8-HxCDF (pg/g-lipid)	1.2	1.6	1.4	1.8
1,2,3,4,6,7,8-HpCDF (pg/g-lipid)	11.7	1.7	13.4	1.9
1,2,3,4,7,8,9-HpCDF (pg/g-lipid)	1.2	2.0	1.2	2.6
OctaCDF (pg/g-lipid)	0.6	2.3	0.7	2.6
TEQ-PCDD/Fs (pg-TEQ/g-lipid)	12.9	1.5	13.4	1.7

N: number of subjects; SD: standard deviation; VNDs: Vietnamese Dongs; BMI: body mass index; *: Geometrical mean and geometrical standard.

**Table 2:** Adjusted comparisons of ADHD scores at 5 years of age between high and low exposure groups.

			Low Exposure					High Exposure			
ADHD-RS	Mean	SD	Adj Mean	95% CI		Mean	SD	Adj Mean	95% CI		*p*-Value
at 5 Years of Age				Lower	Upper				Lower	Upper	
*Boys*											
TCDD	<3 (pg/g lipid) N = 85					≥3 (pg/g lipid) N = 9					
Inattention	30.9	28.1	30.4	24.7	36.1	46.3	26.4	50.4	31.4	69.4	0.052
Hyperactivity	36.8	31.3	36.2	29.9	42.6	54.2	27.7	59.8	38.7	80.8	0.039
ADHD	33.0	29.2	32.5	26.6	38.5	49.3	27.3	54.2	34.5	74.0	0.042
TEQ-PCDD/Fs	<17.6 (pg-TEQ/g lipid) N = 73					≥17.6 (pg-TEQ/g lipid) N = 21					
Inattention	32.3	27.6	32.0	25.6	38.5	32.5	31.1	33.4	20.6	46.2	0.859
Hyperactivity	39.3	30.9	40.4	33.3	47.5	35.8	33.2	31.8	17.6	45.9	0.301
ADHD	35.0	28.5	35.5	28.8	42.2	33.3	32.2	31.3	18.0	44.7	0.593
*Girls*											
TCDD	<3 (pg/g lipid) N = 57					≥3 (pg/g lipid) N = 12					
Inattention	42.0	27.6	42.1	34.7	49.6	51.4	27.9	50.6	33.4	67.8	0.382
Hyperactivity	41.5	32.1	41.0	32.2	49.8	49.8	37.7	52.1	31.8	72.4	0.331
ADHD	41.3	29.2	41.1	33.3	49.0	51.6	31.2	52.4	34.3	70.5	0.270
TEQ-PCDD/Fs	<17.6 (pg-TEQ/g lipid) N = 48					≥17.6 (pg-TEQ/g lipid) N = 21					
Inattention	42.4	28.5	44.3	35.9	52.7	46.3	25.9	42.1	28.8	55.4	0.795
Hyperactivity	43.4	33.0	44.8	34.9	54.7	42.0	34.0	38.7	23.1	54.4	0.537
ADHD	42.4	30.5	44.1	35.2	53.0	44.8	28.0	40.8	26.8	54.9	0.710

N: number of subjects; SD: standard deviation; adj mean: adjusted mean; CI: confidence interval; ADHD-RS: attention deficit hyperactivity disorder rating scale; Adjusted by age, parity, and education of mothers and gestational weeks, birth weight, month age at examination, and family income.

**Table 3:** Adjusted comparisons of ADHD scores at 8 years of age between high and low exposure groups.

	Low Exposure					High Exposure					
ADHD-RS	Mean	SD	Adj Mean	95% CI		Mean	SD	Adj Mean	95% CI		*p*-Value
at 8 Years of Age				Lower	Upper				Lower	Upper	
*Boys*											
TCDD	N = 85					N = 9					
Inattention	43.9	24.1	43.7	38.9	48.6	47.9	18.3	49.1	32.8	65.4	0.538
Hyperactivity	34.0	23.4	33.8	29.0	38.7	32.8	19.3	34.1	13.7	44.6	0.975
ADHD	37.5	21.5	37.3	33.1	41.5	39.4	14.7	40.7	26.6	54.9	0.650
TEQ-PCDD/Fs	N = 73					N = 21					
Inattention	45.2	23.9	44.2	38.8	49.5	41.1	22.8	44.5	33.8	55.1	0.965
Hyperactivity	36.2	22.9	36.2	31.0	41.5	25.5	21.7	25.5	15.1	35.9	0.081
ADHD	39.5	20.9	39.0	34.4	43.6	31.1	20.1	32.8	23.7	41.9	0.246
*Girls*											
TCDD	N = 57					N = 12					
Inattention	53.5	21.1	52.8	46.8	58.9	60.6	25.5	63.5	49.4	77.5	0.181
Hyperactivity	39.1	23.6	39.1	32.8	45.4	56.3	22.5	56.3	41.7	71.0	0.039
ADHD	44.7	21.9	44.3	38.1	50.6	57.6	26.1	59.5	45.0	73.9	0.065
TEQ-PCDD/Fs	N = 48					N = 21					
Inattention	54.2	21.5	53.9	47.0	60.7	55.9	23.1	56.6	45.7	67.4	0.691
Hyperactivity	37.0	22.0	37.2	30.3	44.2	53.8	25.2	53.2	42.2	64.2	0.023
ADHD	44.1	22.4	44.1	37.1	51.1	53.6	23.5	53.5	42.3	64.6	0.181

N: number of subjects; SD: standard deviation; adj mean: adjusted mean; CI: confidence interval; ADHD-RS: attention deficit hyperactivity disorder rating scale; Adjusted by age, parity, and education of mothers and gestational weeks, birth weight, month age at examination, and family income. Cut-off value for TCDD = 3.0 (pg/g lipid); Cut-off value for TEQ-PCDD/Fs = 17.6 (pg-TEQ/g lipid).

**Table 4:** Correlations between ADHD rating scores at 5 years and 8 years of age.

	ADHD-RS at 5 Years of Age												
ADHD-RS	Boys						Girls						
at 8 Years of Age	Inattention		Hyperactivity		ADHD		Inattention		Hyperactivity		ADHD		
*TCDD < 3 (pg/g lipid)*	N = 85						N = 57						
Inattention	0.457	***	0.391	***	0.451	***	0.218		0.195		0.210		
Hyperactivity	0.458	***	0.536	***	0.546	***	0.308	*	0.374	**	0.381	**	
ADHD	0.539	***	0.562	***	0.598	***	0.296	*	0.327	*	0.334	*	
*TCDD ≥ 3 (pg/g lipid)*	N = 9						N = 12						
Inattention	−0.172		−0.522		−0.263		0.004		0.359		0.151		
Hyperactivity	0.160		0.126		0.290		0.258		0.716	**	0.587	*	
ADHD	−0.167		−0.413		−0.114		0.239		0.545		0.451		
*All*	N = 94						N = 69						
Inattention	0.422	***	0.339	***	0.409	***	0.220		0.262	*	0.262	*	
Hyperactivity	0.434	***	0.498	***	0.515	***	0.368	**	0.449	***	0.462	***	
ADHD	0.500	***	0.504	***	0.550	***	0.332	**	0.398	**	0.405	**	

N: number of subjects; ADHD-RS: attention deficit hyperactivity disorder rating scale; *: *p* < 0.05, **: *p* < 0.01, ***: *p* < 0.001.

**Table 5:** Adjusted comparisons of ASRS scores at 5 years of age between high and low exposure groups.

	Low Exposure					High Exposure					*p*-Value
ASRS	Mean	SD	Adj Mean	95% CI		Mean	SD	Adj Mean	95% CI		
at 5 Years of Age				Lower	Upper				Lower	Upper	
*Boys*											
TCDD	N = 83					N = 8					
SC	53.3	7.4	53.4	51.8	55.0	56.9	9.1	55.3	49.8	60.8	0.527
UB	56.6	7.1	56.5	55.1	57.9	58.3	8.4	58.7	53.7	63.6	0.416
DSM	56.4	4.9	56.5	55.4	57.6	60.1	7.4	59.1	55.3	62.8	0.205
TOT	55.4	4.5	55.5	54.5	56.5	58.1	6.3	57.6	54.1	61.0	0.255
TEQ-PCDD/Fs	N = 70					N = 21					
SC	53.3	7.6	53.2	51.4	55.0	54.6	7.9	55.0	51.5	58.5	0.381
UB	56.2	6.8	56.4	54.9	58.0	58.5	8.3	57.6	54.5	60.7	0.529
DSM	56.1	4.9	56.3	55.1	57.5	58.6	6.2	58.0	55.6	60.4	0.225
TOT	55.2	4.5	55.3	54.2	56.3	57.2	5.0	57.0	54.9	59.2	0.157
*Girls*											
TCDD	N = 55					N = 11					
SC	51.8	8.6	51.9	49.5	54.3	54.4	9.9	53.8	48.0	59.5	0.553
UB	57.2	5.0	57.2	55.8	58.6	61.3	6.2	61.3	57.9	64.6	0.031
DSM	55.1	5.4	55.1	53.5	56.7	57.9	8.7	57.7	53.9	61.6	0.229
TOT	54.9	4.8	55.0	53.5	56.4	58.7	7.3	58.2	54.8	61.7	0.089
TEQ-PCDD/Fs	N = 46					N = 20					
SC	53.4	9.1	53.5	50.8	56.1	49.4	7.3	49.1	44.8	53.5	0.110
UB	57.2	5.6	57.3	55.6	58.9	59.5	4.7	59.3	56.6	61.9	0.226
DSM	56.3	6.8	56.4	54.6	58.2	53.8	3.2	53.6	50.7	56.6	0.139
TOT	55.8	6.1	56.0	54.3	57.6	54.8	3.5	54.5	51.8	57.1	0.369

N: number of subjects; SD: standard deviation; adj mean: adjusted mean; CI: confidence interval; ASRS: autism spectrum rating scales; SC: Social communication; UB: Unusual behavior; DSM: Diagnostic and Statistical Manual for Mental Disorders; TOT: Total score; Adjusted by age, parity, and education of mothers and gestational weeks, birth weight, month age at examination, and family income. Cut-off value for TCDD = 3.0 (pg/g lipid); Cut-off value for TEQ-PCDD/Fs = 17.6 (pg-TEQ/g lipid).

**Table 6:** Correlations between ADHD rating scores at 5 and 8 years of age and ASRS scores at 5 years of age (Spearman’s rho).

	ASRS at 5 Years of Age			
ADHD-RS	SC	UB	DSM	TOT
*Boys (N = 91)*				
At 5 years of age				
Inattention	−0.134	0.367 ***	0.143	0.290 **
Hyperactivity	−0.141	0.346 ***	0.067	0.172
ADHD	−0.133	0.375 ***	0.126	0.247 *
At 8 years of age				
Inattention	0.077	0.104	0.173	0.247 *
Hyperactivity	−0.085	0.164	0.045	0.125
ADHD	−0.031	0.196	0.145	0.235 *
*Girls (N = 66)*				
At 5 years of age				
Inattention	−0.176	0.337 **	0.031	0.008
Hyperactivity	−0.244 *	0.310 *	−0.081	−0.039
ADHD	−0.240	0.356 **	−0.045	0.006
At 8 years of age				
Inattention	0.123	−0.061	0.043	0.075
Hyperactivity	−0.216	0.358 **	−0.114	0.041
ADHD	−0.048	0.179	−0.014	0.098

N: number of subjects; ADHD-RS: attention deficit hyperactivity disorder rating scale; ASRS: autism spectrum rating scales; SC: Social communication; UB: Unusual behavior; DSM: Diagnostic and Statistical Manual for Mental Disorders; TOT: Total score; *: *p* < 0.05, **: *p* < 0.01, ***: *p* < 0.001.

## Data Availability

The data presented in this study are available on request to the corresponding author. The data are not publicly available due to the personal information.
